# A Narrative Review of Strategies to Optimize Breastfeeding Among Mothers of Twins

**DOI:** 10.7759/cureus.72792

**Published:** 2024-10-31

**Authors:** Geeta Bhardwaj, Moonjelly Vijayan Smitha

**Affiliations:** 1 College of Nursing, All India Institute of Medical Sciences, Bhopal, Bhopal, IND; 2 College of Nursing, All India Institute of Medical Sciences, Bhubaneswar, Bhubaneswar, IND

**Keywords:** challenges, experience, guidelines, techniques, twins breastfeeding

## Abstract

The global incidence of twin births has substantially increased in recent decades. Twin pregnancies are inherently associated with higher obstetric risks, often resulting in preterm birth and low birth weight, both of which frequently necessitate neonatal intensive care (NICU) interventions. Such interventions can significantly disrupt early breastfeeding practices, contributing to lower exclusive breastfeeding (EBF) rates in twins as compared to singletons. This review examines the factors affecting optimal breastfeeding among twins and strategies for optimization. An extensive literature search was conducted across several databases focusing on breastfeeding twins and related factors. A total of 256 studies on breastfeeding among twins were identified. Additionally, relevant reviews, guidelines, and support materials from twin associations and organizations were examined to identify factors affecting optimal breastfeeding and to highlight strategies for enhancing breastfeeding success. Studies highlight factors affecting optimal breastfeeding among twins, including low milk supply, fatigue, lack of knowledge, anxiety, stress, limited support, breastfeeding reluctance, breast problems, maternal illness, twin prematurity, and NICU admission. Suggested strategies to optimize breastfeeding outcomes include educational support, expressing breastmilk for preterm twins, establishing routines, correct techniques, varied positions, comfort devices, family support, using resources from twin associations, community engagement, and promoting early initiation and EBF. Although factors affecting breastfeeding among twins have been studied, few studies provide strong evidence or statistically determine these issues, and also, interventions are not tailored to individual needs. Studies focusing on personalized interventions are needed to improve breastfeeding outcomes for mothers of twins.

## Introduction and background

Breastfeeding is widely recognized as the optimal source of nutrition for infants, providing essential nutrients for growth and development while offering significant health benefits such as enhanced immune function and reduced risks of chronic conditions later in life [1,2].

Evolving medical advancements and changing lifestyle factors have led to a notable increase in twin pregnancies, primarily attributed to assisted reproductive technologies and fertility treatments [3,4]. Globally, the twinning rate has increased. Evidence from 165 countries by the Human Multiple Birth Database reports the average rate of twin birth as 9-16/1000 in India and 12/1000 worldwide [3,5,6]. Twin pregnancies pose higher risks such as prematurity compared to singletons (71.2% vs. 18.7%) [7,8] and low birth weight, often requiring NICU care, which disrupts breastfeeding and reduces breastfeeding rates [9-12].

The benefits of breastfeeding are multiplied with twins who often are born at risk, such as twins who are often born prematurely with low birth weights, making the immunological and nutritional properties of breast milk crucial for their survival and overall health [1,2]. According to the WHO, optimal breastfeeding involves exclusive breastfeeding (EBF) for the first six months, continued breastfeeding with appropriate complementary foods for up to two years or beyond, and supportive practices to ensure adequate feeding [13]. EBF rates among twins vary from 4% to 22% globally, far less than singletons at 48% [5,14-19]. Breastfeeding twins presents significant challenges similar to those faced by mothers of singletons, visible through the impact on breastfeeding rates and highlighting the need for effective solutions. A critical decision for mothers of twins is whether to breastfeed or bottle-feed. For twin mothers, this choice is further complicated by the demands of caring for multiples. Anecdotal evidence suggests that healthcare professionals and families may discourage breastfeeding for twins. While extensive research supports breastfeeding for singletons, research on optimal breastfeeding practices for twins is limited, with insufficient investigation into the specific factors affecting mothers’ experiences. The lack of scientific evidence complicates the provision of standardized care, contributing to low breastfeeding rates among twins amidst rising multiple births and associated health risks.

This comprehensive narrative review aims to consolidate existing literature on strategies to optimize breastfeeding for twins, addressing the specific factors affecting breastfeeding among twins, and offering practical solutions to promote optimal breastfeeding among twins.

## Review

Search strategy

An extensive literature search was conducted across PubMed, Cochrane Library, Web of Science, Scopus, and Google Scholar, focusing on studies addressing breastfeeding twins, including challenges and solutions. Given the limited studies on twins, the review also considered relevant reviews, guidelines, and support materials. All relevant studies dating back to 1999 were reviewed, along with reference lists, to capture further pertinent information.

A total of 256 studies on breastfeeding challenges were identified using free text and Medical Subject Headings (MeSH) terms, such as breastfeeding twins, fatigue, low milk supply, techniques, preterm, and solutions. Of these, 26 studies focused on breastfeeding twins and were deemed relevant for this comprehensive review. Studies, reviews, and organizational guidelines were explored thoroughly to identify barriers mothers face in achieving optimal breastfeeding for twins.

Results

This review synthesizes maternal experiences, research recommendations, and organizational guidelines to present an evidence-based framework for supporting breastfeeding among twins. Most studies examined breastfeeding practices and rates in twins and multiples, with limited comparison to singleton breastfeeding. A few articles focused on maternal experiences and guidelines for optimizing breastfeeding in multiples, providing insight into best practices.

Optimizing breastfeeding among twins requires identifying and addressing physical, logistic, and psychological barriers, each impacting breastfeeding success. Understanding these challenges and developing targeted solutions can support mothers in feeding twins more effectively, enhancing health outcomes for both mothers and infants.

Key factors hindering breastfeeding success among mothers of twins are given in Table 1.

**Table 1:** Factors affecting breastfeeding among twins

Factors		Categories
Maternal factors	Stress and anxiety due to breastfeeding [12,14,20,21,22].	
	Lack of milk supply [14,15,18,20,22].	
	Exhaustion, fatigue, lethargy [18,20,21,22,23,24].	
	Time-consuming [18,22,23,25- 28].	
	Maternal illness [12,25].	
	Breast issues [22,25,26,27].	
	Unwilling to breastfeed, Mothers' choices and cultural influences [26,27].	
Lack of education and support	Discouragement and lack of support from spouse, family members, and health personnel [11,18,24,27].	
	Lack of knowledge on breastfeeding twins [20,26,28].	
	Obstacles in breastfeeding initiation and continuation- Lack of prenatal education, misinformation and formula feeding in hospital practices [29,30].	
	Challenges in single feeding-Time-consuming sessions, exhaustion, and use of pacifiers often lead to discontinuation of exclusive breastfeeding [22,26].	
	Challenges in Tandem Technique- Privacy concerns, initial support needs, and difficulty burping both twins pose challenges [11,22,27,28,31-33].	
	Difficulty in adopting correct breastfeeding techniques -Mothers lack information on positioning, latching, and simultaneous feeding positions for twins, leading to early cessation [11,22,27-28,31,34].	
	Early discharge and inadequate discharge advices [18].	
Twins factors	The burden of pumping breast milk and then switching to oral feeding becomes challenging for the mother of pre-term twins [18].	
	Infant feeding behavior, prematurity, and NICU admission of twins [21].	

Strategies to Promote Optimal Breastfeeding Among Twins

This review outlines guidelines from literature and breastfeeding organizations to address the challenges effectively. Table 2 highlights the strategies to optimize breastfeeding among twins.

**Table 2:** Strategies to optimize breastfeeding among twins NICU: neonatal intensive care unit References: [11,18,23,25,27,29,31]

Timepoints of strategies implementation	Strategies
Antenatal Phase	Prenatal preparation by education/motivation/ preparing for informed decision-making regarding choice of breastfeeding, techniques, initiation of breastfeeding and steps to overcome initial challenges in breastfeeding twins
Immediately after childbirth	Promoting early initiation of breastfeeding by supporting correct latch, education, and demonstration of correct techniques. Discouraging formula feed and promoting mother’s milk. Motivating and encouraging the mother for her ability to produce sufficient milk for twins.
Birth of Preterm twins	Expression of breast milk and guidelines for storage. Maintenance of the lactation process. Assistance to mothers in the NICU setting.
During hospitalization	Involvement of family members in teaching. Determining family’s role in twin’s breastfeeding.
At administrative level	Administrative steps to support and promote early and exclusive breastfeeding among twins by training health care personnel and abstinence of formula milk and provision of human milk facility near NICU.
At the time of hospital discharge	Tailored need-based discharge advice inclusive of lactational diet and feeding patterns, expression of milk and storage, follow-up, and telephonic interventions. Provision of peer help groups and support groups.

Welcoming multiples presents unique breastfeeding challenges that require systematic support from healthcare professionals for successful lactation. The mother should be well prepared and informed about breastfeeding her twins beforehand. Knowing what to expect ahead of time will make breastfeeding much less complicated and a lot more enjoyable [20].

Several studies have proposed various solutions: education and counseling of mothers during the antenatal and postnatal phases about effective breastfeeding techniques, training of healthcare professionals concerning good positioning, attachment, and effective suckling, and combining discussion with educational materials and practical demonstrations for breastfeeding mothers about positioning and attachment, resulting in significant improvements in effective breastfeeding techniques [11,14,16-18,22-24,29,31-33]. Special attention needs to be given to young, primipara, and poorly educated women.

**Figure 1:**
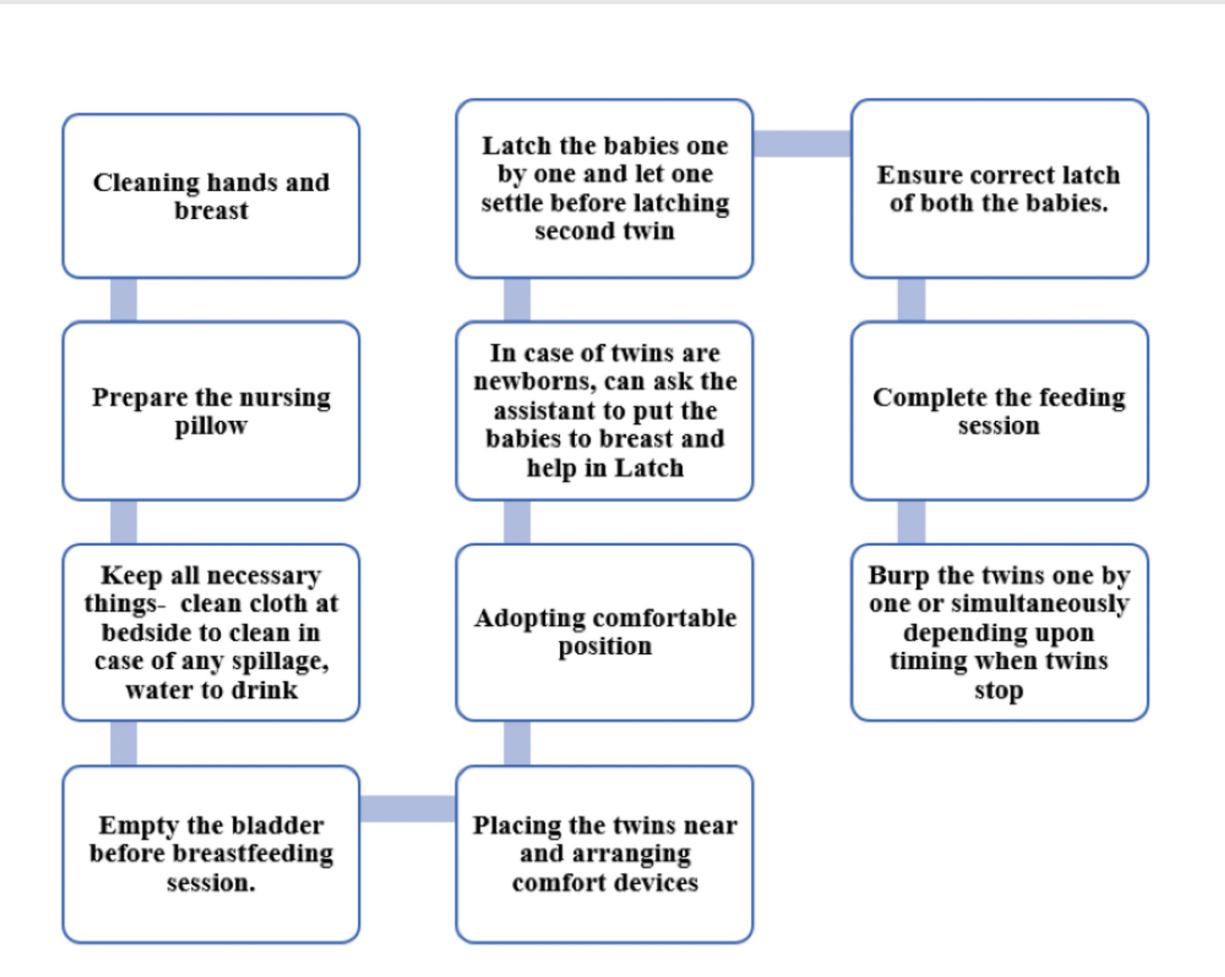
Key steps for an effective breastfeeding session for twins Image Credit: Authors

Creating a supportive breastfeeding environment is essential, particularly for mothers who have undergone cesarean section. Utilizing supportive pillows and armchairs facilitates tandem feeding and enhances comfort. Keeping essentials within reach and ensuring the mother is well-hydrated can further improve the breastfeeding experience. Seeking assistance with latching can also significantly strengthen feeding success [11,23,29]. Figure 1 highlights the key steps for an effective breastfeeding session.

Breastfeeding Education and Techniques

Breastfeeding twins presents unique challenges compared to feeding a single newborn. Supporting mothers of multiples requires specialized education, which can be provided in person, by phone, or online [26]. Specialized interventions tailored to individual needs are critical for facilitating breastfeeding. Mothers may need to adopt simultaneous or staggered feeding approaches to accommodate the distinct demands of each infant. Effective latching is crucial for maternal comfort and optimal feeding, regardless of the chosen position. Mothers should experiment with different breastfeeding positions to determine what works best for them and their infants, as comfort may vary depending on personal preferences and the twins' ages [19,22-24,31,32,35-37].

While breastfeeding one twin at a time allows for individual bonding and may be easier initially, simultaneous feeding can save time once established [19,21]. A flexible feeding schedule often proves beneficial, particularly with a more structured routine during nighttime feedings [19,22].

Many mothers discontinue breastfeeding prematurely due to time constraints, concerns about meeting the needs of both infants, and post-cesarean discomfort. While tandem feeding can reduce the time and effort required for separate sessions, mothers often face discouragement without tailored clinical guidance. Effective breastfeeding techniques are vital to prevent complications, such as cracked nipples and mastitis, which can impact infant growth [17].

Proper positioning and attachment of the infant to the breast are crucial to effective breastfeeding. These techniques enhance milk transfer and minimize nipple trauma and common breastfeeding challenges. Although breastfeeding is a natural process, it is a learned skill, particularly for mothers of twins, who may have specific concerns regarding milk supply and confidence. Mothers should explore various feeding techniques, balancing the trade-offs between efficiency and individual needs [18,27-29].

**Figure 2:**
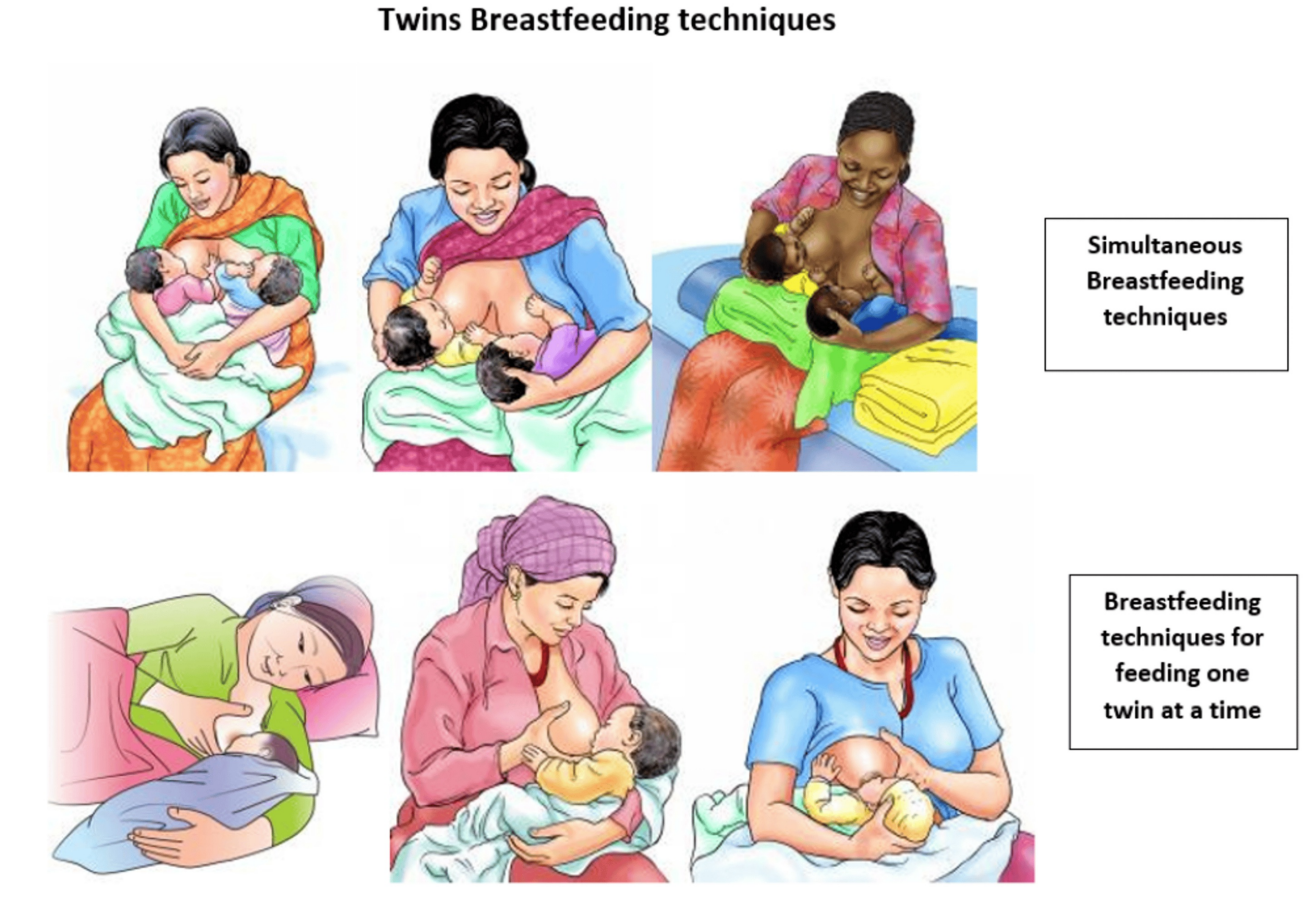
Twins breastfeeding techniques We would like to acknowledge Suaahara1 (https://iycfimagebank.org/organizations/suaahara-I), for the adaptation and use of the images in this review to illustrate the breastfeeding techniques, accessed from the UNICEF IYCF Image Bank (iycfimagebank.org) [41]. Commercial use, redistribution, or selling of these images and materials is prohibited.

Some mothers may prefer one-on-one feeding sessions, while others find simultaneous breastfeeding more practical. Tandem feeding allows simultaneous feeding of both infants, supporting optimal growth and fostering emotional bonds. Research indicates that simultaneous feeding can stimulate milk let-down and support weaker suckling, though it may require assistance to manage proper latching [24,35,36,38-40]. A study highlighted that simultaneous double-pumping resulted in higher prolactin levels, suggesting improved milk yield with frequent use of this technique [38]. Challenges such as lack of privacy and the initial need for support may obstruct mothers from adopting tandem breastfeeding. However, the benefits, time savings, reduced fatigue, and enhanced maternal confidence underscore the importance of targeted interventions and community support to optimize breastfeeding outcomes for twins. Breastfeeding positions for twins are illustrated in Figure 2 [41].

Cradle hold: Common for single breastfeeding, the baby’s head is in the crook of the arm while the body extends along the mother's arm and the body lies close to the mother's abdomen.

Double cradle hold: Both babies are held in cradle position for tandem feeding.

Cross cradle hold: This technique ensures holding the baby across the body with the opposite arm to the nursing breast to ensure better control over the latch. 

Football hold: Babies lie on either side of the mother, feeding on one breast with legs tucked under the mother's arm.

Double-football hold: This technique ensures both babies lie alongside the mother and under arms, heads cradled by the mother's hands and the twins' bodies near the mother's hips.

Laid-back position: The mother lies back, and the baby feeds while lying on the mother's stomach.

Double-laid-back position: The mother lies back and both babies simultaneously lie alongside the mother, either on the stomach or under her arms to breastfeed.

Single side-lying position: The mother and baby lie facing each other, feeding on the bed-side breast.

Upright latch: Babies are held upright in front without crossing over, which is suitable for older babies or those who can sit upright.

Breastfeeding Preterm Twins

Preterm birth due to medical complications, low sucking ability, low birth weight, and NICU admission often leads to maternal-infant separation, hindering breastfeeding initiation. To maintain lactation and provide essential nutritional benefits, mothers should be educated on frequent breast milk expression, techniques, supporting milk supply, and enhancing breastfeeding outcomes by the time the twins start breastfeeding [11,12]. When one twin is in the NICU, mothers can maintain milk supply by expressing milk every two to three hours (8-12 times daily), simulating an infant's feeding pattern, particularly during night hours when lactation hormones peak. NICU facilities often provide pumping rooms with staff assistance, and mothers may consider renting or purchasing a pump if discharged while the baby remains hospitalized. If one twin is home while the other is in the NICU, breastfeeding one twin while expressing milk for the other supports milk production. Staying near the NICU can facilitate skin-to-skin contact through kangaroo mother care and direct feeding if possible, or mothers may use cup feeding with expressed milk when needed [9,40].

Recognizing infant cues and establishing flexible, synchronized feeding patterns are essential for mothers of multiples, enhancing both milk supply and the breastfeeding experience. Research indicates that early, frequent pumping, breast warming, massage, and relaxation can further boost milk production [11,12,18].

In breastfeeding challenges like mastitis, alternating feedings between twins or expressing milk can help maintain flow and support tandem feeding, later removing the perception of low milk supply. A flexible feeding routine, feeding both twins together when possible or individually as needed, can be beneficial, particularly with night feedings in a reclining position to enhance milk supply. Flexibility is critical, as routines adapt to each twin’s cues over time.

Support for Breastfeeding

Effective support for multiple-birth families centers on three fundamental principles: interdisciplinary and community involvement, specialized and coordinated care, and empowering families to make informed decisions [33,34]. Evidence-based practices such as tailored breastfeeding education for families and healthcare providers and a dedicated twin breastfeeding consultation network are essential. Coordinated services, collaborative breastfeeding plans, minimizing mother-infant separation, and support for staggered discharges enhance breastfeeding outcomes and the family’s overall experience [33,34].

Early breastfeeding initiation, ideally right after birth, with assistance from healthcare providers, is recommended, especially following a cesarean section. Many mothers struggle to initiate breastfeeding during hospitalization, highlighting the need for adequate support. Although studies suggest potential solutions, robust evidence from experimental trials is lacking [29,30].

Educational support on twin-specific positioning and latching techniques ensures a comfortable, satisfying breastfeeding experience, which can improve success rates. For twins with differing feeding abilities, breastfeeding the stronger twin while expressing milk to the other is an effective strategy [31].

Many mothers of multiples report insufficient breastfeeding support. For instance, 34% of twin mothers in the United Kingdom and many in Turkey expressed a need for more guidance [16,22]. Studies indicate that mothers of multiples can produce sufficient milk significantly when well-supported [22,39], and educational support can increase breastfeeding rates by up to 74% [19]. Expressing and freezing milk also allows other family members to help with feeding, enabling mothers to focus on breastfeeding while family and friends assist with household and baby care.

Support from community health nurses is needed in terms of awareness, recognition of challenges, improvement of healthcare accessibility, and facilitation of peer support [42,43].

Enhancing maternal confidence and self-efficacy in sustaining milk supply is essential. Mothers must be encouraged to partake nutritious lactational diet to overcome the issue of perceived milk supply. NICU interventions like family-centered care, which involve and educate families, can further improve maternal confidence and breastfeeding success for twins [29]. Workplace accommodations are crucial in maintaining breastfeeding as twins grow [44].

Promoting Breastfeeding While Outdoors With Twins

Mothers often resort to bottle-feeding when outside with their babies due to public barriers such as lack of privacy and social discomfort. However, it's essential to emphasize the importance of breastfeeding in all settings, as it offers unparalleled nutritional and emotional benefits for both mother and child. Mothers should be made aware of unique breastfeeding scarfs designed to provide privacy while breastfeeding in public, reducing discomfort and increasing the ease of nursing twins in public settings. Established breastfeeding facilitates mobility, and advice from experienced mothers emphasizes self-care and avoiding judgmental approaches, crucial elements in successful breastfeeding practices [44].

Recommendations

This review recommends strategies to implement at the hospital, community, and administrative levels to optimize breastfeeding outcomes for mothers of twins.

Premature twins often face increased infection risks, and early breastfeeding is frequently hindered by knowledge gaps, skill deficits, and health issues in mothers and twins. Nurses and midwives are critical in reducing these risks by supporting effective breastfeeding practices in hospital and community settings [11,18].

In hospitals, nurses and midwives can empower mothers of twins by teaching need-based, tailored breastfeeding techniques based on newborn health, gestational age, and weight. Educational sessions can inform families of breastfeeding benefits and involve them in supporting the mother. Antenatal assessments of breast health, cultural practices, and breastfeeding intentions help address challenges early. Nurses and midwives can alleviate fears through demonstrations, videos, and peer experiences, support early tandem breastfeeding, and guide mothers in positioning, latching, recognizing hunger cues, and ensuring sufficient feeding. Additionally, encouraging mothers to log breastfeeding sessions aids in self-assessment, while post-discharge resources, emotional support, and guidance on expressing and storing milk ensure ongoing breastfeeding success, including specialized NICU support when needed [22, 26,27,29,31].

Community-level support is vital to optimize breastfeeding among twins. Nurses and midwives can identify and assess local practices and cultural norms around single versus tandem breastfeeding, recognizing benefits and drawbacks to guide targeted interventions [33]. Educating expectant mothers of twins and their families on the benefits of early initiation, exclusive breastfeeding, and tandem feeding can be effectively achieved through home visits. Additionally, keeping records of breastfeeding practices helps tailor interventions and inform the need for follow-up visits. Family involvement is encouraged to support the mother in feeding techniques, milk expression, and tandem feeding to promote optimal health and development of twins. Mobile video sessions can educate the community on safe breastfeeding practices, correct misconceptions, and encourage healthy behavior changes. Special care for mothers of twins birthing at community health centers and tracking registered expectant mothers ensures ongoing support for optimal breastfeeding outcomes [33,35,43].

Many obstetric nurses and midwives lack experience in supporting mothers who breastfeed twins. To address this, in-service education programs can enhance nurses' and midwives' knowledge and skills in twin breastfeeding care. NICU, in particular, benefits from specialized protocols that cover breastfeeding techniques, milk expression, and storage for ill twins, as well as motivational skills to support mothers with preterm, low birth weight twins, boosting maternal confidence and self-efficacy. Postnatal, antenatal, and outpatient settings can offer classes for twin breastfeeding techniques, while ward managers ensure sufficient nursing support and equipment, like extra pillows, to assist mothers with tandem feeding. Continuing education in lactation counselling specific to twins and special sessions led by lactation counsellors further equips nurses and midwives to guide mothers. Lactation consultants should be available in all antenatal and postnatal settings to optimize breastfeeding for mothers of twins, ensuring improved breastfeeding outcomes. Tracking breastfeeding outcomes with single versus tandem techniques also aids in refining care approaches for twin breastfeeding mothers [11,29,35].

Available Resources to Facilitate Twins' Breastfeeding

Table 3 gives resources to empower parents and families of twins and multiples with guidance, support, and practical tools to assist mothers in effectively breastfeeding their twins, addressing various concerns, providing valuable insights, helping in decision-making, and facilitating a network of community support.

**Table 3:** Breastfeeding resources and organizations for mothers of twins

Associations and organizations/Books	Salient features
Twin Trust [37]	Offers various parent-to-parent support to families of twins through local groups.
Multiple Births Foundation [45]	Provides numerous free leaflets and publications.
BLISS - The Premature Baby Charity [46]	Provides support and guidance for mothers of premature babies.
Australian Breastfeeding Association [47]	Offers booklets on breastfeeding multiples.
Best Beginnings [48]	Provides a DVD, 'Bump to Breastfeeding,' covering topics on twins, expressing, and premature babies.
UNICEF Baby Friendly Initiative [49]	Offers information on baby-friendly care, including leaflets for neonatal units.
Karen Kerkhoff Gromada's 'Mothering Multiples' [50]	A book focusing on breastfeeding multiples.
La Leche League [51]	Provides information on breastfeeding twins via their website.

## Conclusions

Current research on breastfeeding among mothers of twins predominantly focuses on qualitative data, with limited quantifiable data on specific factors influencing breastfeeding practices, highlighting the need for studies to generalize findings and develop effective breastfeeding strategies. Existing solutions for breastfeeding challenges lack statistical validation due to the qualitative nature of information, highlighting the need for targeted trials focused on tailored interventions for mothers of multiples, particularly those with preterm infants, as statistically tested, need-based solutions are essential for evidence-based practice.

However, limited literature has highlighted the need for educational intervention, supportive measures by family and healthcare professionals, and emphasis on correct techniques to optimize twin breastfeeding. Tailored training programs can enhance nurses' and midwives' abilities to provide support, while specific protocols will empower them to teach effective breastfeeding techniques and assist with milk expression for preterm twins. Integrating lactation counseling into ongoing education can further improve outcomes. Collecting comprehensive outcome data is essential to identify best practices for mothers of multiples. These initiatives can enhance support and resources for mothers of twins, promoting better health outcomes for both mothers and their twins.
